# Classifying Muscle States with One-Dimensional Radio-Frequency Signals from Single Element Ultrasound Transducers

**DOI:** 10.3390/s22072789

**Published:** 2022-04-05

**Authors:** Lukas Brausch, Holger Hewener, Paul Lukowicz

**Affiliations:** 1 Fraunhofer Institute for Biomedical Engineering (IBMT), Joseph-von-Fraunhofer-Weg 1, 66280 Sulzbach, Germany; holger.hewener@ibmt.fraunhofer.de; 2 Chair of Embedded Intelligence, Technical University of Kaiserslautern, Gottlieb-Daimler-Straße 47, 67663 Kaiserslautern, Germany; lukowicz@cs.uni-kl.de

**Keywords:** time series classification, ultrasound, radio-frequency signals, machine learning, muscle contractions, muscle fatigue, wearables

## Abstract

The reliable assessment of muscle states, such as contracted muscles vs. non-contracted muscles or relaxed muscles vs. fatigue muscles, is crucial in many sports and rehabilitation scenarios, such as the assessment of therapeutic measures. The goal of this work was to deploy machine learning (ML) models based on one-dimensional (1-D) sonomyography (SMG) signals to facilitate low-cost and wearable ultrasound devices. One-dimensional SMG is a non-invasive technique using 1-D ultrasound radio-frequency signals to measure muscle states and has the advantage of being able to acquire information from deep soft tissue layers. To mimic real-life scenarios, we did not emphasize the acquisition of particularly distinct signals. The ML models exploited muscle contraction signals of eight volunteers and muscle fatigue signals of 21 volunteers. We evaluated them with different schemes on a variety of data types, such as unprocessed or processed raw signals and found that comparatively simple ML models, such as Support Vector Machines or Logistic Regression, yielded the best performance w.r.t. accuracy and evaluation time. We conclude that our framework for muscle contraction and muscle fatigue classifications is very well-suited to facilitate low-cost and wearable devices based on ML models using 1-D SMG.

## 1. Introduction

The reliable assessment of muscle states, such as contracted muscles vs. non-contracted muscles or relaxed muscles vs. fatigue muscles, is very crucial in many sports and rehabilitation scenarios. Signals from various non-invasive and wearable sensors, such as force sensors [1], inertial measurement units (IMUs) [2], mechanomyograms (MMGs) [3,4], surface electromyography (sEMG) [5,6], textile resistive pressure mapping sensors [7] or a combination of those can be used to determine muscle activities or muscle fatigue. More recently, mobile and wearable Electrical Impedance Tomography (EIT) has also been proposed as an imaging method for muscular activities [8].

However, an issue common to all of these techniques is that they only measure signals from the body surface without obtaining information from deeper tissue layers entailing the muscles directly. An alternative non-invasive technique relying on signals extracting information from structures within the body is sonomyography (SMG), which uses ultrasound (US) to obtain information about skeletal muscles. Two-dimensional Brightness mode (B-Mode) US images, which are produced with a US transducer array consisting of several elements, are often used for imaging in SMG-based approaches. A recent review lists 17 studies making use of B-Mode US for biomonitoring muscle and tendon dynamics during locomotion [9]. Even though B-Mode US has shown remarkable accuracies for the classification of various muscle activities and muscle states [9], this technique is not suitable for simple, low-cost and wearable solutions [10].

### 1.1. Related Work

In contrast to two-dimensional images, raw one-dimensional Amplitude Mode (A-Mode) radio frequency (RF) signals can be obtained from comparatively cheap single element US transducers and single channel transmit/receive electronics and do not require any sophisticated processing such as beamforming. A-Mode US is a much better option for low-cost and wearable muscle state recognition solutions and has already been exploited, partially in combination with other sensors, in previous works.

Pioneering work in the field of single element 1-D SMG has been conducted by Guo et. al. for the detection of dynamic thickness changes in skeletal muscles during contractions in 2008 [11] and skeletal muscle assessments for prosthetic controls in 2009 [12]. A follow-up study evaluated the feasibility of signals stemming from single element 1-D SMG for controlling a powered prosthesis with one degree of freedom [13]. In 2013, Machine Learning (ML) algorithms, such as Support Vector Machines (SVMs), and artificial neural networks (ANNs) were used for the first time on single element 1-D SMG signals to predict wrist angles [14]. Muscle fatigue states were first examined with single element 1-D SMG in 2017 by tracking thickness changes of the biceps brachii muscle during the fatigue process [15]. A comparative analysis published in 2018 concluded that the classification performance of US signals acquired with a single element of a conventional linear array transducer exceeded the classification performance of sEMG w.r.t. the recognition of six out of eight hand and wrist gestures but was significantly worse w.r.t. to the recognition of the rest state [16]. Another publication from the same year found that angles and torques of elbows could be reconstructed from 1-D SMG signals using SVM models [17]. The A-Mode US signals of a commercially available system were examined in a 2016 study comprising 206 individuals and it was found that a reliable body fat percentage estimation could be performed on the basis of those signals [18]. In a publication from 2019, A-Mode signals were examined w.r.t. their ability to identify acute changes in muscle thickness after four sets of biceps curls [19]. Another work published in 2020 made use of 1-D SMG signals to measure acoustic nonlinearity parameters and showed that they have the potential to represent skeletal muscles dynamically [20]. An additional publication from 2020 presented a single element wearable ultrasonic sensor, consisting of a transmitter and receiver, and a corresponding method to measure skeletal muscle contractile parameters [21].

### 1.2. Our Contribution

**Figure 1:**
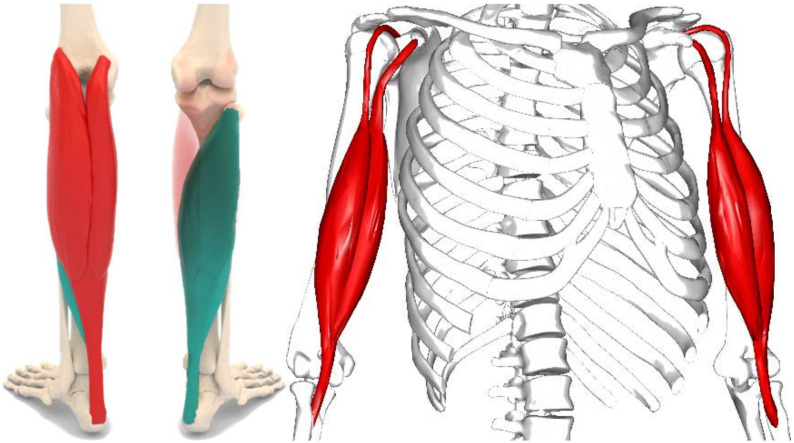
Gastrocnemius muscle (red) and soleus muscle (green) on the left and biceps brachii muscle on the right (Reprinted with permission from [24]. 2008, BodyParts3D [CC BY-SA 2.1 JP]).

The goal of this work was to address the shortcomings of previous approaches by exploiting signals from deeper soft tissue layers that have been acquired with a wearable system, which is only possible with other approaches to a limited extent. To this end, we present a comprehensive ML framework for the classification of 1-D SMG signals stemming from single element US transducers of healthy volunteers to quantify muscle contraction and muscle fatigue states. Our working hypothesis is that it is possible to create ML models discriminating between relaxed and contracted or fatigue signals with high accuracy. In contrast to previous works [11], we did not emphasize the careful selection of any single muscle or muscle group. Instead, we allowed a large degree of freedom w.r.t. the exact US transducer position on a previously defined rough body area, such as the gastrocnemius muscle or biceps brachii, to allow the usage of our methods in environments such as fitness monitoring in gyms or rehabilitation centers. In such environments, we do not expect the user to put any emphasis on transducer positioning for optimal ultrasound signals. Figure 1 illustrates the examined muscles or muscle groups with the gastrocnemius muscle sketched on the left and the biceps brachii muscle sketched on the right. This work builds upon and extends previously published preliminary results [22,23].

## 2. Materials and Methods

### 2.1. Materials

**Figure 2:**
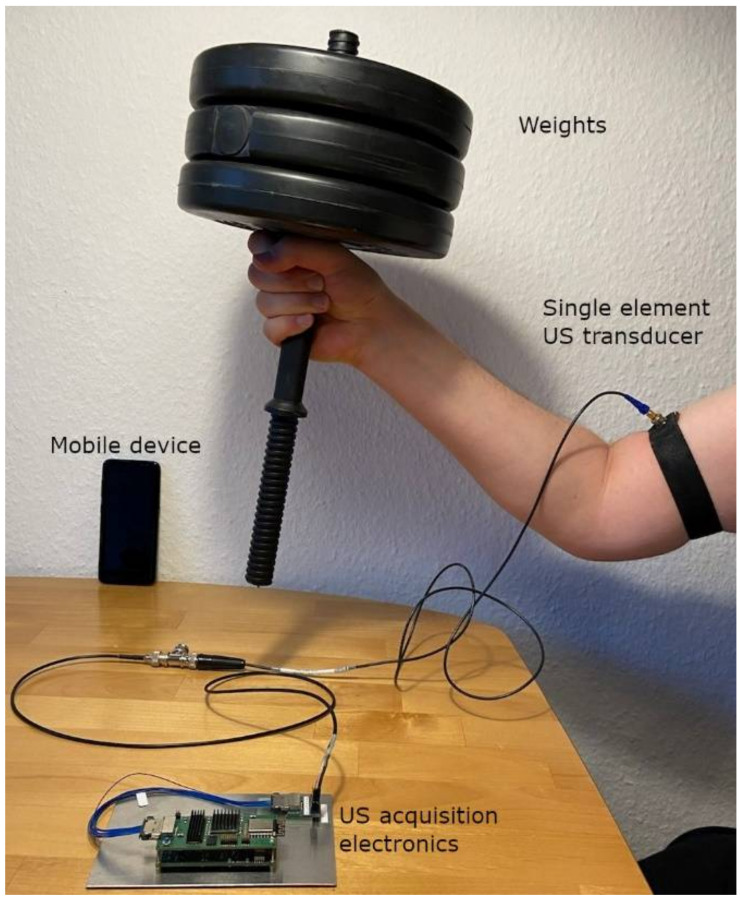
Experimental setup showing a participant lifting a weight, while a single element ultrasound transducer was attached to the body surface via a stretch armband. The signals were acquired with our custom-made acquisition hardware, which transfers them wirelessly to a mobile device.

We relied on 1-D US RF signals of healthy volunteers, which we acquired with the experimental setup shown in Figure 2. It consists of a single element transducer, connected to a custom-designed electronics board (approx. 90 mm × 30 mm × 13 mm), which can be battery powered with a power consumption of approx. 2.5 W while measuring. This board sent the acquired signals of each volunteer via a wireless connection to an Android smartphone with a custom-built app for storage and future processing. We used a Panametrics single element US transducer (with a 3.5 MHz center frequency) to obtain muscle contraction and muscle fatigue data in two different types of experiments.

### 2.2. Methods

Classifying 1-D RF signals poses a time series classification (TSC) task, which is a non-trivial challenge. A publication from 2019 states that TSC “is a hard problem that is not yet fully understood and numerous attempts have been made in the past to create generic and domain-specific classification methods” [25]. We analyzed the signals with a variety of different ML methods and grouped them into traditional ML algorithms, artificial neural networks (ANNs), and Gradient Boosting Machines (GBMs). The traditional ML algorithm was a 1 nearest neighbor approach based on the dynamic time warping distance (1-NN DTW) [26]. The ANNs included a multilayer perceptron (MLP) [27,28], a fully convolutional network (FCN) [27], a radial basis function (RBF) neural network [29,30] and a 1-D residual neural network (ResNet) [27,28]. We also included neural networks using random convolutional kernels. These were ROCKET, MINIROCKET and MultiRocket [31,32,33]. We also included the more recent Transformer model [34,35]. The GBMs included CatBoost [36], LightGBM [37] and XGBoost [38]. We implemented the ANNs in Python with the ML frameworks Keras [30,39] and TensorFlow [40], while we used the DTAIDistance package [41] to deploy 1-NN DTW. We also made use of Scikit-Learn [42] for splitting or standardization of the input data.

To visualize the distribution of the acquired high-dimensional signals and increase our understanding of the signals, we deployed dimensionality reduction techniques (DRTs) such as the linear method Principal Component Analysis (PCA) [43] and the non-linear approach t-distributed stochastic neighbor embedding (t-SNE) [44]. PCA attempts to increase the interpretability of the data by minimizing information loss and maximizing variance. t-SNE converts similarities between data points to joint probabilities and models each high-dimensional object by a low dimensional point in such a way that similar objects are modeled by nearby points and dissimilar objects are modeled by distant points with a higher probability.

### 2.3. Experimental Setup

We acquired signals from eight healthy participants for the muscle contraction classification experiments, in which we asked the subjects to perform squats to distinguish between contracted and non-contracted muscles. We asked all participants to position the US transducer anywhere above the gastrocnemius calf muscle, located on the back of the lower leg (see Figure 1), without instructing them on any specific calf muscle locations. This approach might have resulted in the acquisition of signals suffering from avoidable disturbances, such as interference from neighboring muscles or muscle groups. However, we consider this approach to mimic real-life scenarios, such as usage in a gym or in a rehabilitation facility, adequately.

We acquired data from 21 healthy participants for the muscle fatigue state classification experiments, in which we asked the subjects to lift weights chosen according to the subjectively perceived fitness level for as long as possible to induce muscle fatigue. Muscle fatigue is defined as an exercise-induced reduction in maximal voluntary contraction (MVC) [45]. To simulate real-life scenarios, we did not instruct participants to choose any muscle areas carefully and instead only asked them to put the US transducer on any fitting area above the biceps brachii muscle (see Figure 1). Table 1 and Table 2 provide an overview of the respective gender, total amount of A-scans used for the classification task, total amount of unique datasets, and amount of performed squats or respectively maximum weight lifted for each subject. We provide the raw input data for the muscle contraction experiments [46] and the muscle fatigue experiments [47,48] online. We present the complete database for the muscle contraction classification in Table A1 (Appendix A). Table A2 and Table A3 (Appendix B) show the two respective study designs for the muscle fatigue state classification experiments. All subjects gave their informed consent for inclusion before they participated in the study.

We stored and processed all signals offline on a system suitable to perform the ML classification tasks.

All participants in the muscle contraction study annotated the signals manually by pushing a button during the experiment every time they performed a squat.

For the muscle fatigue signals, we annotated the signals by grouping all signals stemming from the first 10 s of each dataset into the “relaxed” category and all signals stemming from the last 10 s of each dataset into the “fatigue” category. Furthermore, we trimmed the signal sequences by removing the first and last two seconds of each dataset to account for any noise that might have stemmed from lifting or depositing the weight at the beginning and end of each dataset. We conducted two independent studies with the first group (study one) containing signals from all 21 female and male participants, and the second group (study two) containing only signals from a single male subject. This study design allowed us to analyze how the inclusion or exclusion of certain signal types (e.g., signals of subjects with different genders or arm positions) influenced the model performance. The reason for differentiating between genders is that previous small-scale studies have hinted at measurable differences in US B-Mode images of gastrocnemius muscles and tendons during calf raises [49] and differences in shear wave elastography measurements, showing higher passive biceps brachii muscle stiffness in the right arm for women in comparison to men [50]. Study one contains 19,677 annotated A-scans, while study two contains 13,160 annotated A-Scans.

**Figure 3:**
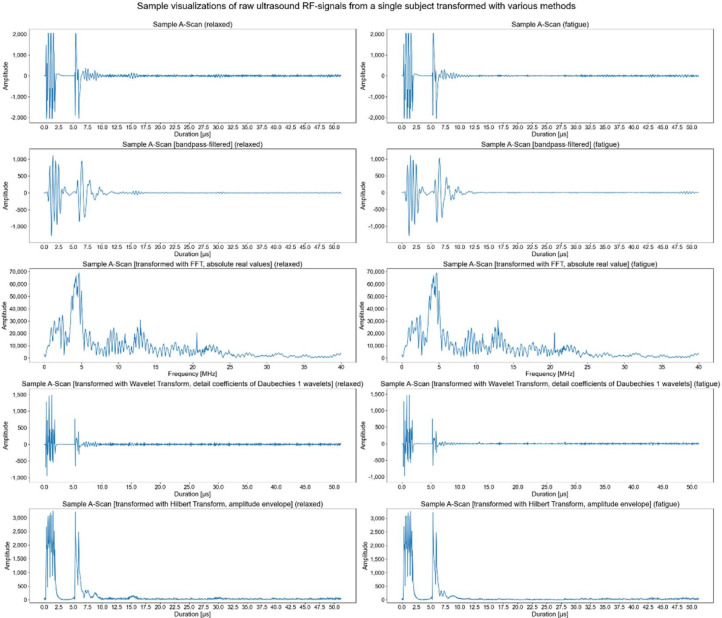
Raw 1-D US RF A-scans and transformed versions for a relaxed and fatigue signal stemming from the same subject.

As we suspected that truncated input data without parts belonging to overdriven excitation signals would lead to better results, we included them in our analysis as well. The raw or truncated signals served as input for our models. These input signals were either not processed at all, pre-processed with a bandpass filter, or transformed with the Fourier transform, Wavelet transform, or Hilbert transform. Figure 3 illustrates the raw 1-D US RF A-scans and transformed versions for a relaxed and fatigue signal stemming from the same subject. Even after careful examination, it is very hard to perceive the very small differences between signals depicted in Figure 3 visually. This necessitates the usage of sophisticated ML models relying on large amounts of data to distinguish between different categories of signals.

Additionally, we also included statistical, spectral, temporal features or a combination thereof in our analysis. We extracted those features with the help of the Time Series Feature Extraction Library for the Python programming language [51].

### 2.4. Evaluation

We only used muscle contraction signals stemming from different datasets from the same person and the same transducer position. For the muscle fatigue data, we distinguished between 12 evaluation modes to compare the impact of a variety of signals and their attributes on the results. Table 3 summarizes the considered evaluation modes.

We divided the available data into testing and training datasets, segregated them according to subject, and desired examined properties and computed the average $F_{1}$-Score to compare the performance for each data type and ML model. For each testing dataset, we used the datasets of all other participants having the desired properties as training data.

**Figure 4:**
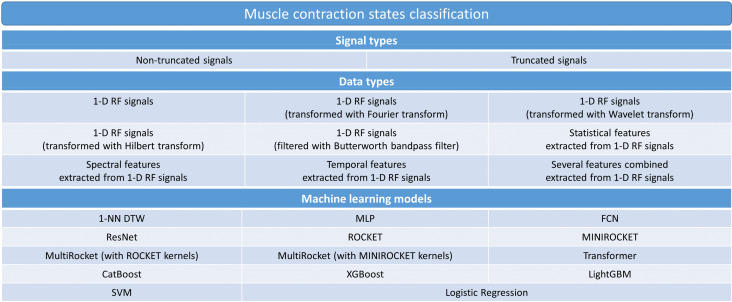
Hierarchical diagram illustrating all computed data input combinations for muscle contraction data.

**Figure 5:**
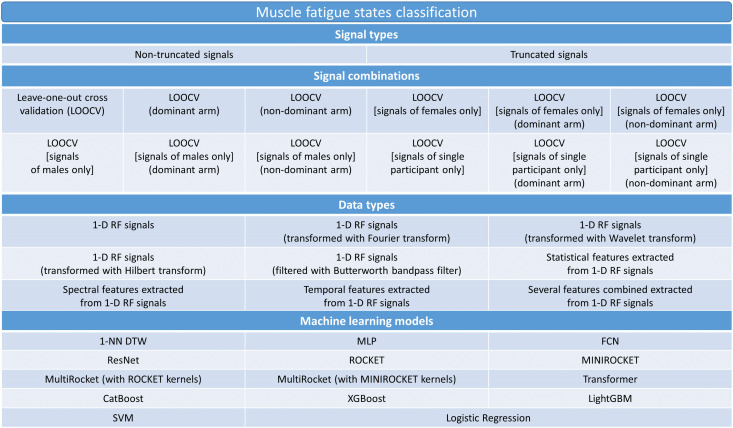
Hierarchical diagram illustrating all computed data input combinations for muscle fatigue data.

For the muscle contraction data classification, we included the ML models MLP, FCN, ResNet, ROCKET, MINIROCKET, MultiRocket, CatBoost, XGBoost, LightGBM, Transformer, 1-NN DTW, SVM and Logistic Regression. For the muscle fatigue data classification, we omitted the models MLP, FCN, and ResNet as their inclusion would have led to a massive increase in computation time by several months for each model, while the expected performance improvement, judging from the results obtained for the muscle contraction classifications, was low [22]. This approach led to 252 possible combinations for the muscle contraction data and 2376 possible combinations for the muscle fatigue data. Figure 4 and Figure 5 show all possible and examined combinations of signal data, data types, and ML models for muscle contraction and muscle fatigue data respectively.

## 3. Results

In this section, we present t-SNE visualizations to gain a better understanding of the high-dimensional distribution of the acquired signals. Those insights can help to interpret the results of the applied ML methods in Section 4 with a thorough discussion of the results. Please note that we omitted the axes of the figures below on purpose, as the t-SNE technique is meant to provide visualizations of the signal distribution and not quantitative results.

### 3.1. Muscle Contraction Signals Classifications

#### 3.1.1. T-Distributed Stochastic Neighbor Embedding

**Figure 6:**
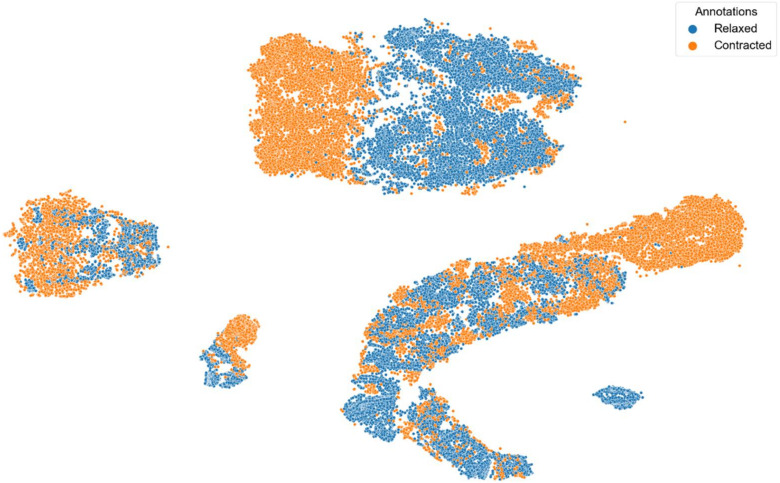
t-SNE visualization illustrating the low-dimensional signal distribution of all datasets with the same transducer position, color-coded according to the muscle state (relaxed vs. contracted). The t-SNE parameters were set to a perplexity of 200, a learning rate of 200, and 10,000 iterations.

**Figure 7:**
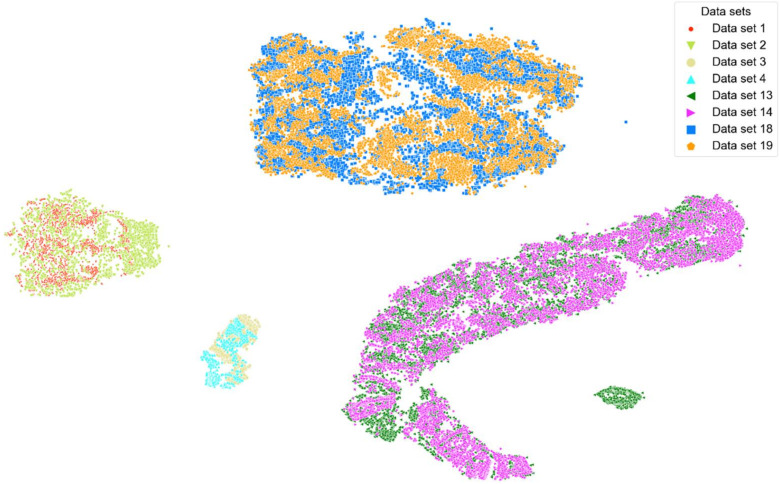
t-SNE visualization illustrating the low-dimensional signal distribution of all datasets with the same transducer position, color-coded according to corresponding datasets. The t-SNE parameters were set to a perplexity of 200, a learning rate of 200, and 10,000 iterations.

Figure 6 and Figure 7 show t-SNE visualizations illustrating the low-dimensional signal distribution of all A-scans from all datasets with the same transducer position. Each dot represents a single A-Scan. Figure 6 is color-coded according to the muscle state (relaxed vs. contracted), while Figure 7 is color-coded according to the datasets the signals stem from. Comparing both figures to each other clearly indicates that the signals group together more strongly according to muscle state than according to the dataset they belong to.

#### 3.1.2. Machine Learning

Table 4 depicts the five best-performing data types and ML model combinations based on the achieved average $F_{1}-$ Scores.

### 3.2. Muscle Fatigue Signals Classifications

#### 3.2.1. T-Distributed Stochastic Neighbor Embedding on Signals of Study One

**Figure 8:**
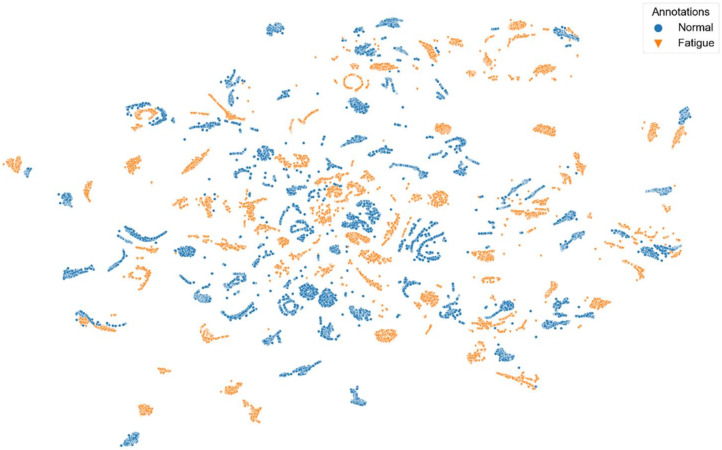
t-SNE visualization illustrating the low-dimensional signal distribution of all datasets from the muscle fatigue study one, color-coded according to the muscle state (normal vs. fatigue). The t-SNE parameters were set to a perplexity of 200, a learning rate of 200, and 10,000 iterations.

**Figure 9:**
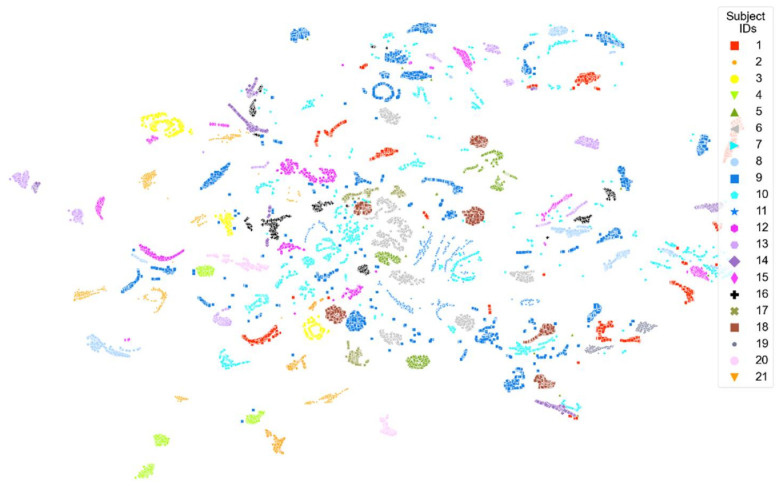
t-SNE visualization illustrating the low-dimensional signal distribution of all datasets from the muscle fatigue study one, color-coded according to corresponding subjects. The t-SNE parameters were set to a perplexity of 200, a learning rate of 200, and 10,000 iterations.

**Figure 10:**
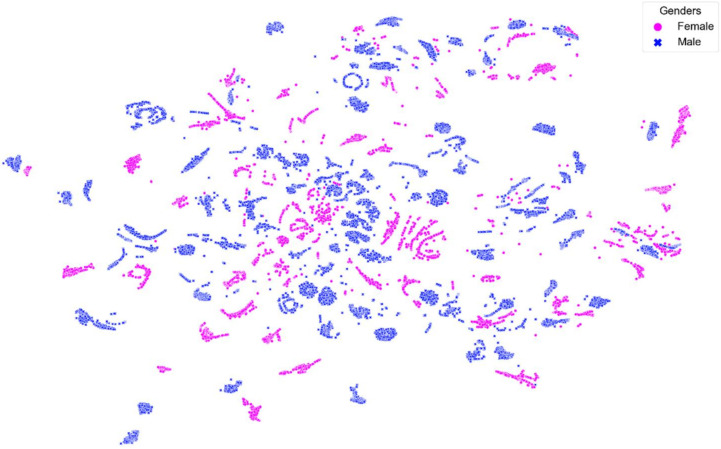
t-SNE visualization illustrating the low-dimensional signal distribution of all datasets with from the muscle fatigue study one, color-coded according to gender (female vs. male). The t-SNE parameters were set to a perplexity of 200, a learning rate of 200, and 10,000 iterations.

**Figure 11:**
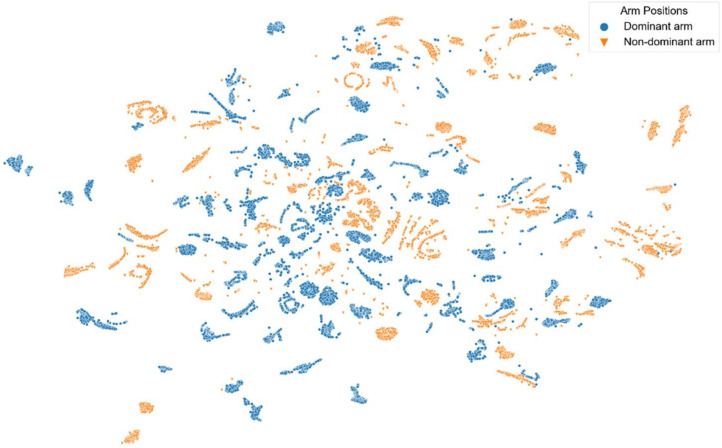
t-SNE visualization illustrating the low-dimensional signal distribution of all datasets from the muscle fatigue study one, color-coded according to the arm position (dominant vs. non-dominant). The t-SNE parameters were set to a perplexity of 200, a learning rate of 200, and 10,000 iterations.

**Figure 12:**
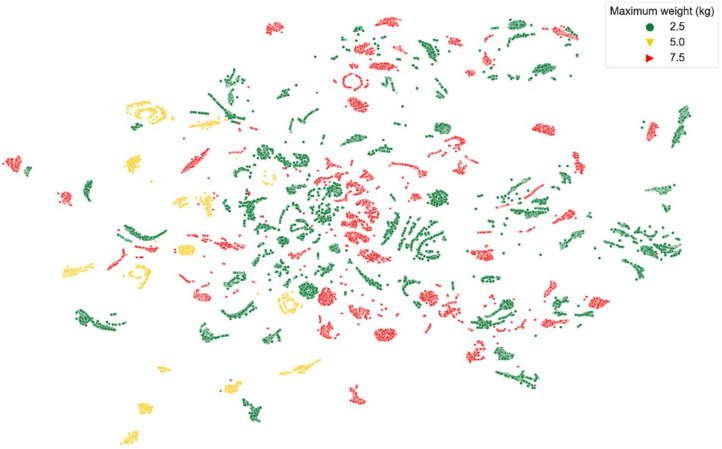
t-SNE visualization illustrating the low-dimensional signal distribution of all datasets from the muscle fatigue study one, color-coded according to the maximum lifted weight (2.5 kg, 5.0 kg, or 7.5 kg). The t-SNE parameters were set to a perplexity of 200, a learning rate of 200, and 10,000 iterations.

Figure 8, Figure 9, Figure 10, Figure 11 and Figure 12 show t-SNE visualizations illustrating the low-dimensional signal distribution of all A-scans from study one. Each dot represents a single A-Scan. Figure 8 is color-coded according to the muscle state (normal vs. fatigue) and Figure 9 is color-coded according to the subjects the signals belong to. Figure 10 is color-coded according to genders (female vs. male), while Figure 11 is color-coded according to the arm the signals stem from (dominant vs. non-dominant). Finally, Figure 12 is color-coded according to the maximum weight lifted by each subject (2.5 kg, 5.0 kg, or 7.5 kg).

Figure 8 shows that there is no strict grouping of the signals according to muscle state, even though a tendency is visible.

Figure 9 shows that the signals have a very strong tendency to group together according to the subject they belong to.

Figure 10 shows that the signals tend to group according to the gender they are annotated with. However, this grouping is not very strict and shows only slight tendencies instead of rigorous borders.

Figure 11 shows that the signals also tend to group according to the arm position they are annotated with. However, this grouping is again not very strict and shows only slight tendencies instead of rigorous borders.

Figure 12 shows a slight tendency of the signals to group according to the maximum weight they have been annotated with.

#### 3.2.2. T-Distributed Stochastic Neighbor Embedding on Signals of Study Two

**Figure 13:**
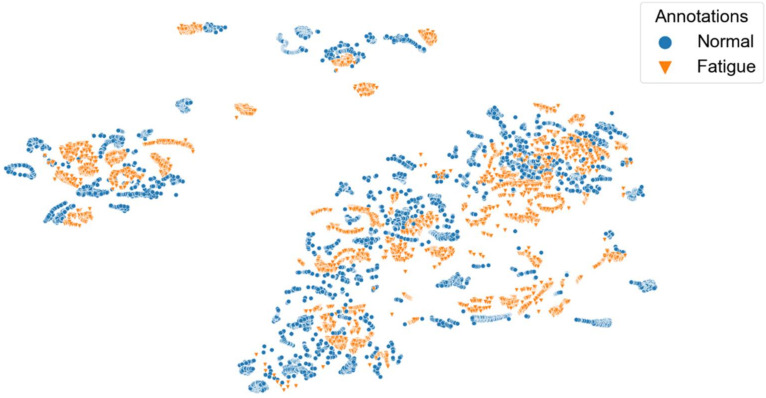
t-SNE visualization illustrating the low-dimensional signal distribution of all datasets from the muscle fatigue study two, color-coded according to the muscle state (normal vs. fatigue). The t-SNE parameters were set to a perplexity of 200, a learning rate of 200, and 10,000 iterations.

**Figure 14:**
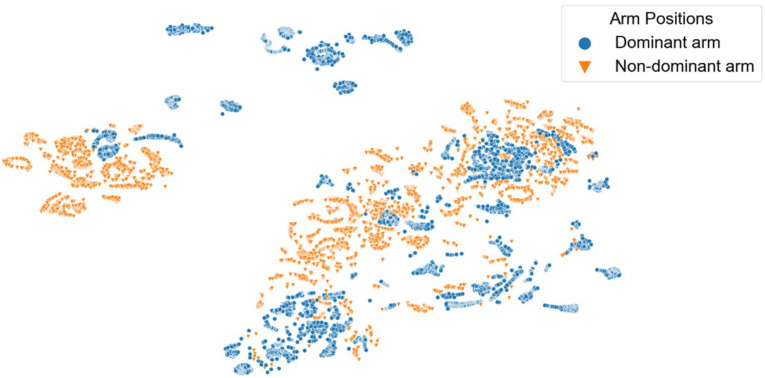
t-SNE visualization illustrating the low-dimensional signal distribution of all datasets from the muscle fatigue study two, color-coded according to the arm position (dominant vs. non-dominant). The t-SNE parameters were set to a perplexity of 200, a learning rate of 200, and 10,000 iterations.

Figure 13 and Figure 14 show t-SNE visualizations illustrating the low-dimensional signal distribution of all A-scans from study two. Each dot represents a single A-Scan. Figure 13 is color-coded according to the muscle state (normal vs. fatigue) and Figure 14 is color-coded according to the arm the signals stem from (dominant vs. non-dominant). In both figures, we removed outliers as they significantly distorted the visual representation.

Figure 13 shows that the signals of study two only have a slight tendency to group according to the muscle state they belong to.

Figure 14 shows that the signals of study two have a strong tendency to group according to the arm position they have been annotated with.

#### 3.2.3. Machine Learning

Table 5 depicts the $F_{1}$ scores and the time needed for training and evaluation of the best performing ML model/data type combinations for the classification of muscle fatigue signals.

A Logistic Regression model relying on extracted spectral features of non-truncated A-scans and an SVM model relying on non-truncated A-Scans, which have been transformed with the Wavelet transform, achieve the best average $F_{1}$ Score of 86%. Both models only require 5 min or less to complete all training and evaluation computations.

## 4. Discussion

### 4.1. Muscle Contraction Signals Classifications

This work only includes results for signals stemming from the same person and the same transducer position for the muscle contraction state classifications. These signals represent 22.55% of all available A-scans, 38.1% of all acquired datasets and entail signals from 37.5% of all subjects. The inclusion of signals stemming from different persons and a variety of transducer positions did not lead to satisfying classification performances. The main reason for this is most probably an insufficient size and diversity of the database. The t-SNE visualizations colored by subjects (see Figure 6) and datasets (see Figure 7) show that the data points cluster stronger according to the subjects they stem from than according to the datasets they belong to. After applying t-SNE, a tendency of the data points to cluster according to their respective annotation was visible but a significant overlap remained (see Figure 6). This tendency allowed us to formulate the hypothesis that models discriminating between relaxed and contracted signals with a high accuracy are possible. An observation is that an SVM model based on Hilbert transformed A-scans achieved an average $F_{1}$- Score of ca. 88% in less than 10 min for training and evaluation. Hence, the SVM model trumped the performance of all ANN models and even 1-NN DTW, which has been the de-facto TSC benchmark for decades [28]. SVM outperforming even the most recent ANNs in terms of speed and accuracy is a remarkable result and paves the way for real-life applications allowing wearable devices to classify different muscle contraction states based on ML models using 1-D SMG signals within minutes.

### 4.2. Muscle Fatigue Signals Classification

The results presented above allow several interpretations of muscle fatigue signal classifications. Firstly, no Gradient Boosting Machine method ranked among the top-performing models. Another observation is that using the model 1-NN DTW did not result in any competitive results, even though this algorithm has been the de-facto TSC benchmark for decades [28]. Model 1-NN DTW was prohibitively slow for the scenarios described in this work. The convolutional neural networks based on the ROCKET family [31,32,33] could achieve competitive results but always performed worse than SVM and Logistic Regression w.r.t. accuracy and time, regardless of the underlying data types used. The relatively straight-forwarded algorithms SVM and Logistic Regression always performed better than other algorithms and even outperformed complex deep learning approaches. These models are also, on average, among the fastest approaches. The deep learning Transformer models, that adopt the mechanism of attention, are the most recent ML architectures used in this work. Those models yielded competitive results in many scenarios but were never able to outperform all other models in any case. Transformers also required a prohibitive amount of time for training and evaluation.

SVM and Logistic Regression were superior approaches for all muscle fatigue state classification evaluation strategies. ML models based on raw A-scans usually outperformed models based on truncated A-Scans. Regardless of the best performing data type or model, the approaches based on signals of the dominant arm always outperformed approaches based on the non-dominant arm or signals of both arms in a mixed setting. Models based on all signals of only female subjects performed slightly worse than models based on all signals of only male subjects. A possible explanation is that less data was available for female subjects. An additional notable observation is that models based on signals of a single male subject performed, on average, worse than models based on different subjects. A possible explanation is that the inclusion of many diverse signals from subjects with different training levels allowed for easier signal discriminations in comparison to comparatively homogeneous signals stemming from a single subject with no change in training level. The training and evaluation times of the best-performing models always stayed below one hour for models based on signals of a certain arm. Only the best performing model based on all signals, mixing signals from the dominant and non-dominant arm, needed several hours to finish training and evaluation. These results show that scenarios requiring a near real-time classification of muscle fatigue states based on 1-D SMG signals are possible.

### 4.3. Future Work

Even though average $F_{1}$- Scores ranging from 70% to 88% would not qualify the presented algorithms for critical clinical use, they would still be suitable for use cases in sports–and rehabilitation scenarios as mentioned above. We expect that improved performances will result from the availability of more data in the future. Real-life implementations might include libraries such as LIBSVM [52] to deploy the proposed framework directly on mobile devices. Using cloud computing resources might also be a solution for more complex models when these are trained and evaluated remotely, while the local mobile devices would simply acquire the signals without processing them any further in that scenario.

Additionally, alternative ML models might be used in the future to obtain even better accuracies. For example, TSC approaches based on transfer learning [53] have shown promising results in the past. Further work might also exploit data fusion from multiple modalities, as shown in previous works by others [54]. Adding more data, such as accelerometer measurements, age, or body mass index, could also be a solution to create more sophisticated ML models.

By openly releasing the datasets for this work [46,47,48], we encourage others to build on our work and hope to inspire further research in this promising field.

## 5. Conclusions

To the best of our knowledge, this work presents, for the first time, the implementation and evaluation of a unified framework for the classification of 1-D US RF signals of muscle contraction and muscle fatigue states. This is a crucial step towards mobile and wearable solutions, which could find applications in rehabilitation or fitness tracking scenarios. To this end, we simulated real-life scenarios as closely as possible by not asking participants to emphasize obtaining particularly distinct signals and not examining the skin surface with B-Mode US first to find strongly pronounced muscle areas.

We find that the amount, quality and annotation strategies of our data allow to build robust, accurate, and fast models. Even though training and initial evaluation of these models requires a significant amount of time and computational power, the inference computations, yielding results by presenting previously unseen signals to the trained models, merely require milliseconds to complete.

Very complex and sophisticated ML models are not necessary to obtain reasonably robust models. The straight-forward and well-tried algorithms SVM and Logistic Regression always outperform more sophisticated and complex approaches, such as ANNs, Gradient Boosting Machines, or 1-NN DTW.

## Figures and Tables

**Table 1:** Muscle contraction signals database (summary).

Subject ID	Gender	# A-Scans	# Datasets	# Squats
1	Male	92,000	7	154
2	Male	2000	2	8
3	Female	18,872	2	35
4	Male	20,000	2	49
5	Male	10,000	1	27
6	Male	10,000	1	21
7	Male	20,000	2	88
8	Male	40,000	4	133

**Table 2:** Muscle fatigue signals database (summary).

Subject ID	Gender	# A-Scans	# Datasets	Max. Weight [kg]
01	Female	18,283	4	5.0
02	Female	15,155	3	2.5
03	Male	16,161	2	2.5
04	Male	18,863	2	2.5
05	Male	4302	2	7.5
06	Male	27,112	4	7.5
07	Female	13,585	4	5.0
08	Male	8809	3	5.0
09	Male	109,964	51	7.5
10	Female	7967	3	5.0
11	Female	3326	2	5.0
12	Male	4349	2	5.0
13	Male	14,691	3	7.5
14	Female	6950	2	5.0
15	Male	12,817	2	5.0
16	Male	10,218	2	5.0
17	Male	3792	1	7.5
18	Male	14,005	3	5.0
19	Male	5236	2	7.5
20	Male	4635	2	7.5
21	Female	11,817	2	2.5

**Table 3:** Evaluation modes for muscle fatigue signal classifications.

Evaluation Mode	Study	Signals Taken from Study [%]
Leave-one-out cross-validation (LOOCV) on all signals	1	100
LOOCV on signals from dominant arm only	1	52.48
LOOCV on signals from non-dominant arm only	1	47.52
LOOCV on signals from female subjects only	1	33.44
LOOCV on signals from male subjects only	1	66.56
LOOCV on signals from dominant arm of female subjects only	1	17.54
LOOCV on signals from non-dominant arm of female subjects only	1	17.67
LOOCV on signals from dominant arm of male subjects only	1	34.94
LOOCV on signals from non-dominant arm of male subjects only	1	31.62
LOOCV on signals from a single subject only	2	100
LOOCV on signals from dominant arm of a single subject only	2	54.89
LOOCV on signals from non-dominant arm of a single subject only	2	45.11

**Table 4:** Muscle contraction signals classification results (summary).

Model	Data Type	Signals Truncated	$\mathrm{Average}\;F_{1}-\;\mathrm{Score}\;\mathrm{for}\;\mathrm{All}\;\mathrm{Data}\;\mathrm{Types}\;[\%]$	Standard Deviation for All Data Types	Time for Training and Evaluation [h]	$\mathrm{Average}\;F_{1}-\mathrm{Score}\;[\%]$
SVM	Hilbert transformed A-Scans	no	85	1.95	0.17	88
MLP	Hilbert transformed A-Scans	no	84	1.97	6.66	88
SVM	Fourier transformed A-Scans	no	85	1.95	0.09	87
MLP	Fourier transformed A-Scans	no	84	1.97	6.12	87
SVM	Wavelet transformed A-Scans	no	85	1.95	0.12	86

**Table 5:** Muscle fatigue signals classification results (summary).

Evaluation Mode	ML Model	Data Type	$F_{1}\mathrm{Score}\;(\%)$	Time for Evaluation and Training (Minutes)
LOOCV	SVM	Wavelet transformed A-scans	82	334
LOOCV (dominant arm)	SVM	Wavelet transformed A-scans	84	20
LOOCV (non-dominant arm)	SVM	Combination of all possible features	77	<5
LOOCV (female)	Logistic Regression	Combination of all possible features	77	<5
LOOCV (female) [dominant arm]	Logistic Regression	Spectral features	86	<5
LOOCV (female) [non-dominant arm]	Logistic Regression	Wavelet transformed A-Scans	75	<5
LOOCV (male)	SVM	Wavelet transformed A-scans	84	50
LOOCV (male) [dominant arm]	SVM	Wavelet transformed A-scans	86	5
LOOCV (male) [non-dominant arm]	SVM	Combination of all possible features (of truncated signals)	79	<5
LOOCV (single subject 09)	Logistic Regression	Statistical features	70	<5
LOOCV (single subject 09) [dominant arm]	SVM	Wavelet transformed A-scans	78	7
LOOCV (single subject 09) [non-dominant arm]	SVM	Temporal features	72	<5

## Data Availability

Publicly available datasets were analyzed in this study. This data can be found in [46,47,48].

## Appendix A

Table A1 shows the complete database of all signals acquired for the muscle contraction classification experiments. Eight healthy volunteers (7 male, 1 female) performed squats to induce muscle contractions. The volunteers placed the US transducer either in a unique position anywhere above the gastrocnemius calf muscle or kept the transducer in place for the acquisition of further datasets, which is indicated by non-unique transducer positions. Additionally, we also present the total amount of acquired A-scans and the average amount of A-scans acquired per second for each dataset.

**Table A1:** Complete muscle contraction signals database.

Dataset ID	Subject ID	Gender	Unique Transducer Position	# A-Scans	A-Scans/s
1	01	male	no	3000	54.71
2	01	male	no	3000	50.55
3	02	male	no	1000	55.32
4	02	male	no	1000	52.06
5	01	male	yes	6000	56.17
6	01	male	yes	50,000	33.95
7	03	female	yes	10,000	48.56
8	03	female	yes	8872	56.17
9	04	male	yes	10,000	48.65
10	04	male	yes	10,000	38.98
11	05	male	yes	10,000	40.82
12	06	male	yes	10,000	45.33
13	07	male	no	10,000	58.73
14	07	male	no	10,000	59.55
15	01	male	yes	10,000	59.97
16	01	male	yes	10,000	60.33
17	01	male	yes	10,000	60.19
18	08	male	no	10,000	47.58
19	08	male	no	10,000	47.48
20	08	male	yes	10,000	54.44
21	08	male	yes	10,000	44.88

## Appendix B

Table A2 and Table A3 show the two respective study designs for the muscle fatigue classification experiments. A total of 21 healthy volunteers (14 male, 7 female) lifted weights to induce an MVC resulting in muscle fatigue. The volunteers placed the US transducer on a unique position anywhere above the biceps brachii muscle of the dominant or non-dominant arm. We present the genders, the amount of datasets, the total amount of A-scans used for the classification task, and the maximum lifted weights for each subject. The maximum lifted weight was chosen according to the subjectively perceived fitness level of each test subject. For some subjects, the weights were adjusted in later datasets.

**Table A2:** Muscle fatigue signals database for all subjects (study one).

Subject ID	Gender	# Datasets	# A-Scans	Max. Lifted Weight [kg]
01	female	4	1390	5.0
02	female	3	1043	2.5
03	male	2	696	2.5
04	male	2	685	2.5
05	male	2	695	7.5
06	male	4	1386	7.5
07	female	4	1367	5.0
08	male	3	1044	5.0
09	male	10	3453	7.5
10	female	3	1044	5.0
11	female	2	696	5.0
12	male	2	695	5.0
13	male	3	1035	7.5
14	female	2	695	5.0
15	male	2	666	5.0
16	male	2	672	5.0
17	male	1	348	7.5
18	male	3	1035	5.0
19	male	1	342	7.5
20	male	1	345	7.5
21	female	1	345	2.5

**Table A3:** Muscle fatigue signals database for a single subject (study two).

Subject ID	Gender	# Datasets	# A-Scans	Max. Lifted Weight [kg]
09	male	42	13,160	7.5
